# Change Point Detection in Panel Linear Regression Models Based on Jump Information Criterion

**DOI:** 10.3390/e28040375

**Published:** 2026-03-26

**Authors:** Wenzhi Zhao, Lu Fan, Zhiming Xia

**Affiliations:** 1 School of Science, Xi’an Polytechnic University, Xi’an 710048, China220811034@stu.xpu.edu.cn (L.F.); 2 School of Mathematics, Northwest University, Xi’an 710127, China

**Keywords:** change point, panel data, jump information criterion, convergence rate

## Abstract

This paper focuses on the critical issue of change point detection in panel linear regression models and proposes a novel jump information criterion (JIC) for efficient solution. The core innovation of this criterion lies in reconstructing the traditional change point hypothesis testing problem into a parameter estimation problem: under the null hypothesis ($H_{0}$, i.e., no change point exists in the model) and the alternative hypothesis ($H_{1}$, i.e., a change point exists in the model), the number of potential change points is set to 0 and 1 for modeling and solution, respectively. To verify the theoretical reliability of the proposed method, this paper systematically establishes the consistency of the change point count estimator through rigorous mathematical deductions and further derives its optimal convergence rate. In terms of numerical validation, extensive Monte Carlo simulation experiments and real data empirical analysis both demonstrate that the estimator constructed based on JIC exhibits excellent performance in change point identification accuracy, stability, and computational efficiency, providing a reliable new tool for structural break analysis in panel data models.

## 1. Introduction

Panel data models constitute a pivotal analytical tool for exploring multi-dimensional datasets, with a conventional assumption that model parameters remain constant across the entire observation period. In practical scenarios, however, this parameter stability is frequently disrupted by external factors such as policy adjustments, economic fluctuations, or unforeseen public events, which may induce structural changes in model parameters at unknown time points. To ensure the reliability of empirical modeling, it is therefore essential to test for the existence of such change points in panel data and accurately estimate their locations if they exist, an issue that has spurred extensive methodological research in the field. The methodological exploration of change point detection in panel data traces its origins to Joseph and Wolfson (1992, 1993) [1,2], who laid the foundational theoretical framework for this research area. Since then, substantial advancements have been made through diverse methodological approaches. In the realm of parameter estimation, Bai (2010) [3] proposed a least squares method to identify common mean change points in panel data with stationary error terms, establishing a cornerstone for parameter-based change point analysis. In terms of hypothesis testing, Horváth (2012) [4] and Chen and Hu (2017) [5] both adopted the cumulative sum (CUSUM) approach to detect mean change points, while Li et al. (2015) [6] extended this line of research to variance change point detection by developing a squared CUSUM method.

As research has advanced, scholars have increasingly focused on addressing more complex panel data structures and heterogeneities. Li, Qian, and Su (2016) [7] examined panel models with interactive fixed effects and proposed a penalized principal component procedure integrated with adaptive group fused LASSO to detect multiple structural breaks, supporting their method with rigorous theoretical justifications and comprehensive simulation and empirical validations. Baltagi et al. (2017) [8] further generalized change point estimation to encompass both stationary and nonstationary panel data contexts, systematically deriving the asymptotic properties of least squares and first-difference estimators. For high-dimensional panel data characterized by a large number of cross-sections and short time series, Jaromír et al. (2018) [9] designed a Wald-type test to enhance detection power.

Recent studies have also made significant strides in tackling complex error structures and heterogeneous panel settings. Baltagi et al. (2020) [10] investigated drift tests for time-trend panel models with serially correlated error components, proposing a Wald-type test statistic based on the fixed-effects feasible generalized least squares (FE-FGLS) estimator and confirming its limiting distribution as a chi-square distribution. Okui and Wang (2021) [11] developed a method combining grouped fixed effects and adaptive grouped fused LASSO to estimate structural breaks in heterogeneous panel models, while Karim and Nase (2022) [12] introduced the Double-CUSUM-Modified EWMA method to improve the sensitivity of mean shift detection in panel data. Lumsdaine, Ryo, and Wang (2023) [13] focused on linear panel models with grouped heterogeneity, proposing a least squares approach to jointly estimate break points, group memberships, and model coefficients. Most recently, Wang, Phillips, and Su (2024) [14] addressed the challenges posed by interactive fixed effects and unobserved heterogeneities (including latent group structures and unknown structural breaks) in linear panel models, proposing a binary segmentation method for break point estimation and a sequential testing K-means algorithm to identify latent group structures and their numbers.

In the aforementioned literature, change point analysis is typically conducted in two sequential steps: detection followed by estimation. While these methods have demonstrated promising performance in addressing change point detection problems, most of them necessitate assumptions regarding certain relevant quantities or rely on predefined threshold values. Furthermore, a number of current approaches have been developed under the premise that the sequences of random variables conform to specific distributions. In practical applications, however, data distributions tend to be highly diverse. Consequently, although many methods exhibit sound theoretical properties, their utility in real-world scenarios remains constrained.

In scenarios where prior information about the sample is unavailable, Xia and Qiu (2015) [15] proposed the jump information criterion (JIC) for estimating discontinuous nonparametric regression curves. This criterion comprises two key components: a fitting term that aligns with the observed data and a penalty term associated with the complexity introduced by change points. The number of change points is determined by minimizing the JIC value. In this study, we extend the application of the JIC method to detect change points in panel regression models. Specifically, we transform the change point detection problem into an estimation problem centered on determining whether the number of change points is one or zero. Moreover, if a change point exists, its location is estimated concurrently. A notable advantage of this method is that it neither depends on critical values nor requires the error sequence to follow specific distributions. The approach presented in this paper integrates the traditional two-step framework of change point analysis detection and estimation into a unified methodological framework. This integrated design mitigates error accumulation, improves computational efficiency, and exhibits stronger adaptability to complex real-world contexts.

The remainder of this paper is structured as follows. Section 2 outlines the model framework and fundamental assumptions. Section 3 elaborates on our proposed methodology and presents the core results, including a rigorous demonstration of the consistency of the estimates and their corresponding convergence rates. Section 4 reports the results of Monte Carlo simulations and an empirical case study. Finally, Section 5 summarizes the key findings of the paper and provides concluding remarks.

## 2. Model and Assumptions

### 2.1. The Model

Consider the following regression panel data model

(1) $$y_{it}=a_{i}+x_{i,t}^{\prime }\left(\beta +\Delta I\left\{t>k^{*}\right\}\right)+\varepsilon_{i,t},\;i=1,\cdots ,N,t=1,\cdots ,T,$$

where $x_{i,t}$ is a p×1 vector of regressors, β is a p×1 vector of unknown coefficients, $\varepsilon_{i,t}$ is the error term with zero mean, and $a_{i}$ is the individual fixed effects. We consider the testing problem of whether a change point exists. The regression coefficients in the panel data may change from β to β+Δ at an unknown time $k^{*}$, which is usually referred to as a change point or structural break. Accordingly, model (1) can be rewritten as

(2) $$y_{it}=\left\{\begin{array}{cc} a_{i}+x_{it}^{\prime }\beta_{1}+\varepsilon_{it}, & t=1,\ldots ,k^{*},\\ a_{i}+x_{it}^{\prime }\beta_{2}+\varepsilon_{it}, & t=k^{*}+1,\ldots ,T, \end{array}\;i=1,\ldots ,N,\right.$$

where $\beta_{2}=\beta_{1}+\Delta$.

We propose a jump information criterion (JIC) for solving at most one change-point problem in panel data. Under the null hypothesis, the coefficients do not change, while under the alternative hypothesis, the coefficients undergo a change. Under the null hypothesis, there is no change in the coefficients, while under the alternative hypothesis, there is a change in the coefficients, i.e., the null hypothesis and alternative hypothesis are stated as

$$H_{0}:∥\Delta ∥=0\;↔\;H_{1}:∥\Delta ∥\neq 0,$$

where ∥·∥ denotes the Euclidean norm of vectors and matrices.

### 2.2. Assumptions

For the subsequent theoretical results, the following assumptions on the model are required:

**Assumption 1.** 

(i) *The errors $\varepsilon_{i}={\left(\varepsilon_{i,1},\varepsilon_{i,2},\ldots ,\varepsilon_{i,T}\right)}^{\prime },i=1,\ldots ,N$ are independent;*
(ii) *$E\varepsilon_{i,t}=0,E\varepsilon_{i,t}\varepsilon_{i,s}=0$ and $c_{1}\leq \sigma^{2}=E\varepsilon_{i,t}^{2}\leq c_{2}$ for all 1≤i≤N,1≤t≠s≤T with some constants $0<c_{1}\leq c_{2}<\infty$.*
(iii) *The ith observation satisfies the strict exogeneity condition, that is, $E\left(\varepsilon_{i,t}|x_{i,1},\cdots ,x_{i,T}\right)=0$ which ensures that the demeaned error term $\left(\varepsilon_{i,t}-{\bar{\varepsilon }}_{i}\right)$ is uncorrelated with the demeaned regressors $\left(x_{i,t}-{\bar{x}}_{i}\right)$.*

**Assumption 2.**  *If change point $k^{*}$ exists, then $\pi^{*}\in \left[e,1-e\right]$ satisfies*

$$\frac{k^{*}}{T}\to \pi^{*}\;\mathrm{as}\;T\to \infty .$$

**Remark 1.**  *The breakpoint position $k^{*}$ depends on the sample size T, and the two are approximately proportional, where e is a small positive constant.*

**Assumption 3.**  *There is $c_{3}$ such that $∥x_{i,t}∥\leq c_{3}$ for all 1≤i≤N,1≤t≤T.*

**Assumption 4.** 

(i) *There are positive constants $k_{1}$ and $c_{4}$ such that ${\left(\sum_{j=1}^{k_{1}}x_{i,j}x_{i,j}^{\prime }\right)}^{-1}$ exists and $∥{\left(\sum_{j=1}^{k_{l}}x_{i,j}x_{i,j}^{\prime }\right)}^{-1}∥\leq c_{4}$ for all i,1≤i≤N;*
(ii) *There exist positive constants $k_{2}$ and $c_{5}$ such that ${\left(\sum_{j=T-k_{2}}^{T}x_{i,j}x_{i,j}^{\prime }\right)}^{-1}$ exists and*
*$∥{\left(\sum_{j=T-k_{2}}^{T}x_{i,j}x_{i,j}^{\prime }\right)}^{-1}∥$$\leq c_{5}$ for all i,1≤i≤N.*

Note that Assumption 4 (i) implies $∥{\left(\sum_{j=1}^{k_{l}}x_{i,j}x_{i,j}^{\prime }\right)}^{-1}∥\leq c_{4}$ for all $k\geq k_{1}$. Similarly, as in Assumption 4 (ii), we find that $∥{\left(\sum_{j=k+1}^{T}x_{i,j}x_{i,j}^{\prime }\right)}^{-1}∥\leq c_{5}$ for all $k\leq T-k_{2}$. Assumption 4 (i) ensures that the OLS estimators in (2) are uniquely defined.

## 3. Methodology and Main Results

### 3.1. Methodology

First, we introduce some notation that will be used throughout the paper. Since we are dealing with detection and estimation of an unknown change point, we will analyze various estimators, matrices, and parameters before and after a certain time k,1≤k≤T.

Let

$${\bar{y}}_{i}=\frac{1}{T}\sum_{t=1}^{T}y_{it},\;{\bar{x}}_{i}=\frac{1}{T}\sum_{t=1}^{T}x_{it},$$

be the mean of *y* and x, respectively, for the *i*th individual. The least squares estimates of β and $a_{i}$ in the model (1) are given by

$${\hat{\beta }}_{w}=W_{xx}^{-1}W_{xy},\;{\hat{a}}_{i}={\bar{y}}_{i}-{\hat{\beta }}_{w}^{\prime }{\bar{x}}_{i},\;i=1,\cdots ,N,$$

where

$$\begin{array}{c} W_{xx,i}=\sum_{t=1}^{T}\left(x_{it}-{\bar{x}}_{i}\right){\left(x_{it}-{\bar{x}}_{i}\right)}^{\prime },\\ W_{xy,i}=\sum_{t=1}^{T}\left(x_{it}-{\bar{x}}_{i}\right)\left(y_{it}-{\bar{y}}_{i}\right),\\ W_{yy,i}=\sum_{t=1}^{T}{\left(y_{it}-{\bar{y}}_{i}\right)}^{2}. \end{array}$$

Let $W_{xx}=\sum_{i=1}^{N}W_{xx,i},W_{xy}=\sum_{i=1}^{N}W_{xy,i},W_{yy}=\sum_{i=1}^{N}W_{yy,i}$; the residual sum of squares of (1) is

$$S_{NT}=W_{yy}-{\hat{\beta }}_{w}^{\prime }W_{xx}{\hat{\beta }}_{w}.$$

When Model (1) contains at most one change point, let ${\hat{\beta }}_{1k}$ and ${\hat{\beta }}_{2k}$ denote the OLS estimators computed from the first *k* and the last T−k observations of the panel model, respectively. Hence,

(3) $$\begin{array}{cc} \; & {\hat{\beta }}_{1k}={\left(W_{xx}^{1}\right)}^{-1}W_{xy}^{1},\\ \; & {\hat{\beta }}_{2k}={\left(W_{xx}^{2}\right)}^{-1}W_{xy}^{2}, \end{array}$$

where $W_{xx}^{1}=\sum_{i=1}^{N}\sum_{t=1}^{k}\left(x_{it}-{\bar{x}}_{i}\right){\left(x_{it}-{\bar{x}}_{i}\right)}^{\prime },W_{xx}^{2}=\sum_{i=1}^{N}\sum_{t=k+1}^{T}\left(x_{it}-{\bar{x}}_{i}\right){\left(x_{it}-{\bar{x}}_{i}\right)}^{\prime }$, $W_{yy}^{1}=\sum_{i=1}^{N}\sum_{t=1}^{k}{\left(y_{it}-{\bar{y}}_{i}\right)}^{2},$ and $W_{yy}^{2}=\sum_{i=1}^{N}\sum_{t=k+1}^{T}{\left(y_{it}-{\bar{y}}_{i}\right)}^{2}.$

In this paper, the parameter estimation problem can be formulated as the following optimization problem:

(4) $$\left({\hat{\beta }}_{1},{\hat{\beta }}_{2},{\hat{k}}^{*}\right)=\underset{p<k<T,\beta_{1k},\beta_{2k}}{\arg\min}\left(W_{yy}^{1}-\beta_{1}^{\prime }W_{xx}^{1}\beta_{1}+W_{yy}^{2}-\beta_{2}^{\prime }W_{xx}^{2}\beta_{2}\right),$$

where $W_{yy}^{1}=\sum_{i=1}^{N}\sum_{t=1}^{k}{\left(y_{it}-{\bar{y}}_{i}\right)}^{2},W_{yy}^{2}=\sum_{i=1}^{N}\sum_{t=k+1}^{T}{\left(y_{it}-{\bar{y}}_{i}\right)}^{2}$.

The estimation of (4) is conducted in three steps:

Step 1: Given p<k<T−p, obtained ${\hat{\beta }}_{1k},{\hat{\beta }}_{2k}$:
  (5) $$\left({\hat{\beta }}_{1k},{\hat{\beta }}_{2k}\right)=\underset{\beta_{1k},\beta_{2k}}{\arg\min}\left(W_{yy}^{1}-\beta_{1k}^{\prime }W_{xx}^{1}\beta_{1k}+W_{yy}^{2}-\beta_{2k}^{\prime }W_{xx}^{2}\beta_{2k}\right)$$
Step 2: Obtained $\hat{k}$:
  (6) $$\begin{array}{cc} \hat{k}= & \underset{p<k<T-p}{\arg\min}\left(W_{yy}^{1}-{\hat{\beta }}_{1k}^{\prime }{\hat{W}}_{xx}^{1}{\hat{\beta }}_{1k}+W_{yy}^{2}-{\hat{\beta }}_{2k}^{\prime }{\hat{W}}_{xx}^{2}{\hat{\beta }}_{2k}\right)\\ \; & =\underset{p<k<T-p}{\arg\min}S_{Nk} \end{array}$$
Step 3: Estimate $\beta_{1},\beta_{2},\Delta :$
  (7) $${\hat{\beta }}_{1}={\hat{\beta }}_{1\hat{k}},{\hat{\beta }}_{2}={\hat{\beta }}_{2\hat{k}},\hat{\Delta }={\hat{\beta }}_{2}-{\hat{\beta }}_{1}$$

We propose a jump information criterion (JIC) for addressing the problem of detecting at most one change point in panel data, with a specific focus on testing for shifts in regression parameters. The panel-data-adaptive JIC is defined as follows:

(8) $$\mathrm{JIC}\left(m\right)=\frac{\mathrm{SSR}(m)}{NT}+P\left(N,T\right)\cdot \sum_{j=1}^{m}\frac{1}{{\left(N\cdot ∥{\hat{\Delta }}_{j}∥\right)}^{\gamma }},\;m=0,1,$$

where JIC comprises two components. The first component $\frac{\mathrm{SSR}(m)}{NT}$ is referred to as the fitting term, measuring the model’s goodness of fit. The second component is a penalty term composed of P(NT) and ${\left(N\cdot ∥\hat{\Delta }∥\right)}^{-\gamma }$, considering the magnitude of jumps at change points. γ≥0 serves as a tuning parameter, and P(N,T) in the second term acts as an adjustment factor to ensure that JIC(m) penalizes increment more than the reduction in the sum of squared residuals under $H_{0}$ and penalizes increment less than the reduction in the sum of squared residuals under alternative hypothesis.

Notations are defined as follows:

$$\mathrm{SSR}\left(m\right)=\left\{\begin{array}{cc} S_{Nk}, & m=1,\\ S_{NT}, & m=0. \end{array}\right.$$

Then, (8) can be rewritten as follows:

$$\begin{array}{cc} & \mathrm{JIC}\left(0\right)=\frac{S_{NT}}{NT},\\ & \mathrm{JIC}\left(1\right)=\frac{S_{Nk}}{NT}+P\left(N,T\right)\cdot \frac{1}{(N\cdot ∥\hat{\Delta }{∥)}^{\gamma }}. \end{array}$$

By minimizing the JIC(m), an estimate $\hat{m}$ of the true number of change points $m_{0}$ can be obtained, which facilitates the determination of whether change points exist:

(9) $$\hat{m}=\underset{m\in \{0,1\}}{\arg\min}\mathrm{JIC}\left(m\right).$$

If $\hat{m}=0$, it indicates the estimation of no change points. Otherwise, there exists a change point, and in this case, the estimated change point location is ${\hat{k}}^{*}$.

### 3.2. Main Results

Define the ideal objective function ${\mathrm{JIC}}^{*}\left(m\right)$ as a deterministic approximation of JIC(m). Taking the limit of the random component of JIC(m) while keeping its non-random part unchanged yields ${\mathrm{JIC}}^{*}\left(m\right)$ as a deterministic function. If $m_{0}=0$, then,

$${\mathrm{JIC}}^{*}\left(m\right)=\left\{\begin{array}{cc} \sigma^{2}, & m=0,\\ \sigma^{2}+P\left(N,T\right)\cdot O_{p}{\left(\frac{\sqrt{T}}{N}\right)}^{\gamma }, & m=1. \end{array}\right.$$

If $m_{0}=1$, then,

$${\mathrm{JIC}}^{*}\left(m\right)=\left\{\begin{array}{cc} \sigma^{2}+\frac{{\left(\beta_{1}-\beta_{2}\right)}^{\prime }{\left[{\left(W_{xx}^{1}\right)}^{-1}+{\left(W_{xx}^{2}\right)}^{-1}\right]}^{-1}\left(\beta_{1}-\beta_{2}\right)}{NT}, & m=0,\\ \sigma^{2}+P\left(N,T\right)\cdot (N\cdot ∥\hat{\Delta }{∥)}^{-\gamma }, & m=1. \end{array}\right.$$

We need the following assumption:

**Assumption 5.**  *Given the range of values of P(N,T):*

$$0<P\left(N,T\right)<\frac{{\left(\beta_{1}-\beta_{2}\right)}^{\prime }{\left[{\left(W_{xx}^{1}\right)}^{-1}+{\left(W_{xx}^{2}\right)}^{-1}\right]}^{-1}\left(\beta_{1}-\beta_{2}\right)}{NT}\cdot {\left(N\cdot ∥\Delta ∥\right)}^{\gamma }.$$

**Proposition 1.**  *Given Assumptions 1–4 and Assumption 5, $m_{0}$ is the unique minimizer of the ideal objective function ${\mathrm{JIC}}^{*}\left(m\right)$.*

**Proof.**  When $m_{0}=0,$ it can be known from Assumption 5 that P(N,T)>0, so

$$\begin{array}{cc} {\mathrm{JIC}}^{*}\left(1\right)-{\mathrm{JIC}}^{*}\left(0\right)= & P\left(N,T\right)\cdot O_{p}\left({\left(\frac{\sqrt{T}}{N}\right)}^{\gamma }\right)\\ & >0\cdot O_{p}\left({\left(\frac{\sqrt{T}}{N}\right)}^{\gamma }\right)\\ & =0. \end{array}$$

Thus, when $m_{0}=0$, ${\mathrm{JIC}}^{*}\left(0\right)<{\mathrm{JIC}}^{*}\left(1\right)$.

When $m_{0}=1$, according to Assumption 5, we know that

$P\left(N,T\right)<\frac{{\left(\beta_{1}-\beta_{2}\right)}^{\prime }{\left[{\left(W_{xx}^{1}\right)}^{-1}+{\left(W_{xx}^{2}\right)}^{-1}\right]}^{-1}\left(\beta_{1}-\beta_{2}\right)}{NT}\cdot {\left(N\cdot ∥\Delta ∥\right)}^{\gamma }$, from which it follows that

$$\begin{array}{cc} & {\mathrm{JIC}}^{*}\left(1\right)-{\mathrm{JIC}}^{*}\left(0\right)\\ & =P\left(N,T\right)\cdot {\left(N∥\Delta ∥\right)}^{-\gamma }-\frac{{\left(\beta_{1}-\beta_{2}\right)}^{\prime }{\left[{\left(W_{xx}^{1}\right)}^{-1}+{\left(W_{xx}^{2}\right)}^{-1}\right]}^{-1}\left(\beta_{1}-\beta_{2}\right)}{NT}\\ & <\frac{{\left(\beta_{1}-\beta_{2}\right)}^{\prime }{\left[{\left(W_{xx}^{1}\right)}^{-1}+{\left(W_{xx}^{2}\right)}^{-1}\right]}^{-1}\left(\beta_{1}-\beta_{2}\right)\cdot {\left(N\cdot ∥\Delta ∥\right)}^{\gamma }}{NT}\cdot {\left(N\cdot ∥\Delta ∥\right)}^{-\gamma }\\ & -\frac{{\left(\beta_{1}-\beta_{2}\right)}^{\prime }{\left[{\left(W_{xx}^{1}\right)}^{-1}+{\left(W_{xx}^{2}\right)}^{-1}\right]}^{-1}\left(\beta_{1}-\beta_{2}\right)}{NT}\\ & =0. \end{array}$$

Therefore, ${\mathrm{JIC}}^{*}\left(1\right)<{\mathrm{JIC}}^{*}\left(0\right)$ when $m_{0}=1$. □

**Proposition 2.**  *Under Assumptions 1–4 and Assumption 5, we have*

(10) $$\underset{m\in \{0,1\}}{\sup}\left|\mathrm{JIC}\left(m\right)-{\mathrm{JIC}}^{*}\left(m\right)\right|=O\left(P\left(N,T\right)\right),\;\;a.s.\;$$

**Proof.**  Let $D\left(m\right)=\mathrm{JIC}\left(m\right)-{\mathrm{JIC}}^{*}\left(m\right)$. If $m_{0}=0$, then

(11) $$D\left(m\right)=\left\{\begin{array}{cc} \frac{S_{NT}}{NT}-\sigma^{2}, & m=0,\\ \frac{S_{N\hat{k}}}{NT}-\sigma^{2}+P\left(N,T\right)\cdot \left[\left((N∥\hat{\Delta }{∥)}^{-\gamma }-O_{p}{\left(\frac{\sqrt{T}}{N}\right)}^{\gamma }\right)\right], & m=1. \end{array}\right.$$

From (11) it follows that:

(12) $$\begin{array}{cc} \; & \underset{m\in \{0,1\}}{\sup}\left|D\left(m\right)\right|\\ \; & =\left|\frac{S_{N\hat{k}}}{NT}-\sigma^{2}+P\left(N,T\right)\cdot \left[(N∥\hat{\Delta }{∥)}^{-\gamma }-O_{p}\left({\left(\frac{\sqrt{T}}{N}\right)}^{\gamma }\right)\right]\right|\\ \; & =O(P(N,T)),\;a.s.\; \end{array}$$

If $m_{0}=1$, we then have

(13) $$D\left(m\right)=\left\{\begin{array}{cc} \frac{S_{NT}}{NT}-\sigma^{2}-\frac{{\left(\beta_{1}-\beta_{2}\right)}^{\prime }{\left[{\left(W_{xx}^{1}\right)}^{-1}+{\left(W_{xx}^{2}\right)}^{-1}\right]}^{-1}\left(\beta_{1}-\beta_{2}\right)\cdot {\left(N\cdot ∥\Delta ∥\right)}^{\gamma }}{NT}, & m=0,\\ \frac{S_{N\hat{k}}}{NT}-\sigma^{2}+P\left(N,T\right)\cdot \left[(N∥\hat{\Delta }{∥)}^{-\gamma }-{\left(N∥\Delta ∥\right)}^{-\gamma }\right], & m=1. \end{array}\right.$$

Similarly, it follows from (13) that

(14) $$\begin{array}{cc} \; & \underset{m\in \{0,1\}}{\sup}\left|D\left(m\right)\right|\\ = & \left|\frac{S_{N\hat{k}}}{NT}-\sigma^{2}+P\left(N,T\right)\cdot \left[(N∥\hat{\Delta }{∥)}^{-\gamma }-{\left(N∥\Delta ∥\right)}^{-\gamma }\right]\right|\\ = & O(P(N,T)),\;a.s.\; \end{array}$$

□

**Theorem 1.**  *For all P(N,T) satisfying Assumption 5 and any ε>0, we have*

(15) $$\lim_{(N,T)\to \infty }P\left(h_{N,T}\left(\hat{m}-m_{0}\right)<\varepsilon \right)=1$$

*where $h_{N,T}=\frac{K_{N,T}}{P(N,T)}$ and*

$$K_{N,T}=\;\min\left\{P\left(N,T\right)\cdot O_{p}\left({\left(\frac{\sqrt{T}}{N}\right)}^{\gamma }\right),\frac{{\left(\beta_{1}-\beta_{2}\right)}^{\prime }{\left[{\left(W_{xx}^{1}\right)}^{-1}+{\left(W_{xx}^{2}\right)}^{-1}\right]}^{-1}\left(\beta_{1}-\beta_{2}\right)}{NT}-P\left(N,T\right)\cdot {\left(N∥\Delta ∥\right)}^{-\gamma }\right\}.$$

**Proof.**  According to Proposition 2, we can find a set *A* such that $P\left(A\right)\overset{P}{\to }1$, and for all ω∈A, (10) holds. The functions D(m) and JIC(m) share the same minimizer $\hat{m}$. Furthermore, from (11) and (13), given any neighborhood

$$W_{NT,\varepsilon }=\left(m_{0}-\varepsilon \frac{P(N,T)}{K_{N,T}},m_{0}+\varepsilon \frac{P(N,T)}{K_{N,T}}\right),$$

where D(m) follows Proposition 2,

$$K_{N,T}=\;\min\left\{P\left(N,T\right)\cdot O_{p}\left({\left(\frac{\sqrt{T}}{N}\right)}^{\gamma }\right),\frac{{\left(\beta_{1}-\beta_{2}\right)}^{\prime }{\left[{\left(W_{xx}^{1}\right)}^{-1}+{\left(W_{xx}^{2}\right)}^{-1}\right]}^{-1}\left(\beta_{1}-\beta_{2}\right)}{NT}-P\left(N,T\right)\cdot {\left(N∥\Delta ∥\right)}^{-\gamma }\right\}.$$

there exists a constant $d_{NT}>0$ such that

(16) $$\begin{array}{cc} d_{N,T} & =\underset{W_{NT,\varepsilon }^{c}}{\inf}{\mathrm{JIC}}^{*}\left(m\right)-{\mathrm{JIC}}^{*}\left(m_{0}\right)\\ \; & =\left\lceil \varepsilon \frac{P(N,T)}{K_{N,T}}\right\rceil \left[K_{N,T}+o\left(K_{N,T}\right)\right]\\ \; & =\varepsilon P\left(N,T\right)+\varepsilon P\left(N,T\right)o\left(1\right)+\left(\left\lceil \varepsilon \frac{P(N,T)}{K_{N,T}}\right\rceil -\varepsilon \frac{P(N,T)}{K_{N,T}}\right)\left[K_{N,T}+o\left(K_{N,T}\right)\right]\\ \; & \geq \varepsilon P(N,T). \end{array}$$

According to the definition of ${\mathrm{JIC}}^{*}\left(m\right)$, we have $o\left(K_{N,T}\right)>0$, where ⌈x⌉ denotes the smallest integer not less than *x*.

On the other hand, according to Proposition 2 and the definition of $\hat{m}$, for ω∈A, there always exists a positive integer n=n(ω) such that when T>n, we obtain

(17) $$\begin{array}{cc} \; & {\mathrm{JIC}}^{*}\left(\hat{m}\right)-{\mathrm{JIC}}^{*}\left(m_{0}\right)\\ \; & =\left({\mathrm{JIC}}^{*}\left(\hat{m}\right)-\mathrm{JIC}\left(\hat{m}\right)\right)+\left(\mathrm{JIC}\left(\hat{m}\right)-\mathrm{JIC}\left(m_{0}\right)\right)+\left(\mathrm{JIC}\left(m_{0}\right)-{\mathrm{JIC}}^{*}\left(m_{0}\right)\right)\\ \; & \leq \left|{\mathrm{JIC}}^{*}\left(\hat{m}\right)-\mathrm{JIC}\left(\hat{m}\right)\right|+\left(\mathrm{JIC}\left(\hat{m}\right)-\mathrm{JIC}\left(m_{0}\right)\right)+\left|{\mathrm{JIC}}^{*}\left(m_{0}\right)-{\mathrm{JIC}}^{*}\left(m_{0}\right)\right|\\ \; & <\varepsilon P(N,T)/2+0+\varepsilon P(N,T)/2=\varepsilon P(N,T). \end{array}$$

Combining (16) and (17), we have

$${\mathrm{JIC}}^{*}\left(\hat{m}\right)<\underset{W_{NT,\varepsilon }^{c}}{\inf}{\mathrm{JIC}}^{*}\left(m\right),T>n,\omega \in A.$$

This implies that when $T>n,\omega \in A,\hat{m}\in W_{NT,\varepsilon }$. Thus, Theorem 1 is proved. □

Theorem 1 indicates that $\hat{m}$ is consistent and converges to $m_{0}$ at a rate of $1/h_{N,T}$. In this case, the following corollary can be derived:

**Corollary 1.**  *Under Assumptions 1–5, when*

*$P\left(N,T\right)=\frac{{\left(\beta_{1}-\beta_{2}\right)}^{\prime }{\left[{\left(W_{xx}^{1}\right)}^{-1}+{\left(W_{xx}^{2}\right)}^{-1}\right]}^{-1}\left(\beta_{1}-\beta_{2}\right)\cdot {\left(N\cdot ∥\Delta ∥\right)}^{\gamma }}{NT\left[N^{-\gamma }T^{\frac{\gamma }{2}}\cdot {\left(N\cdot ∥\Delta ∥\right)}^{\gamma }+1\right]}$, the estimator $\hat{m}$ converges to $m_{0}$ at the optimal rate of $O{\left(\frac{\sqrt{T}}{N}\right)}^{-\gamma }$.*

**Proof.**  According to the definition of $h_{N,T}$ in Theorem 1,

$$\begin{array}{cc} h_{N,T} & =\frac{K_{N,T}}{P(N,T)}\\ & =\min\left\{O_{p}{\left(\frac{\sqrt{T}}{N}\right)}^{\gamma },\frac{{\left(\beta_{1}-\beta_{2}\right)}^{\prime }{\left[{\left(W_{xx}^{1}\right)}^{-1}+{\left(W_{xx}^{2}\right)}^{-1}\right]}^{-1}\left(\beta_{1}-\beta_{2}\right)}{NT\cdot P(N,T)}-{\left(N∥\Delta ∥\right)}^{-\gamma }\right\}. \end{array}$$

To maximize $h_{N,T}$, we have

$$N^{-\gamma }\cdot T^{\frac{\gamma }{2}}=\frac{{\left(\beta_{1}-\beta_{2}\right)}^{\prime }{\left[{\left(W_{xx}^{1}\right)}^{-1}+{\left(W_{xx}^{2}\right)}^{-1}\right]}^{-1}\left(\beta_{1}-\beta_{2}\right)}{NT\cdot P(N,T)}-{\left(N∥\Delta ∥\right)}^{-\gamma }.$$

Hence,

$$P\left(N,T\right)=\frac{{\left(\beta_{1}-\beta_{2}\right)}^{\prime }{\left[{\left(W_{xx}^{1}\right)}^{-1}+{\left(W_{xx}^{2}\right)}^{-1}\right]}^{-1}\left(\beta_{1}-\beta_{2}\right)\cdot {\left(N\cdot ∥\Delta ∥\right)}^{\gamma }}{NT\left[N^{-\gamma }T^{\frac{\gamma }{2}}\cdot {\left(N\cdot ∥\Delta ∥\right)}^{\gamma }+1\right]}.$$

In this case, we can obtain that $h_{N,T}$ is

$$\begin{array}{cc} h_{N,T} & =\frac{K_{N,T}}{P(N,T)}\\ & =\min\left\{O_{p}\left({\left(\frac{\sqrt{T}}{N}\right)}^{\gamma }\right),\frac{{\left(\beta_{1}-\beta_{2}\right)}^{\prime }{\left[{\left(W_{xx}^{1}\right)}^{-1}+{\left(W_{xx}^{2}\right)}^{-1}\right]}^{-1}\left(\beta_{1}-\beta_{2}\right)}{NT\cdot P(N,T)}-{\left(N∥\Delta ∥\right)}^{-\gamma }\right\}\\ & =O_{p}\left({\left(\frac{\sqrt{T}}{N}\right)}^{\gamma }\right). \end{array}$$

As a result, the convergence rate of $\hat{m}$ is $O{\left(\frac{\sqrt{T}}{N}\right)}^{-\gamma }$, and Corollary 1 is proved. □

**Corollary 2.**  *Under Assumption 1 and Theorem 1 hold, we have as (N,T)→∞*

(18) $$\lim_{(N,T)\to \infty }P\left(\hat{m}=m_{0}\right)=1.$$

**Proof.**  Since ε in Theorem 1 can be arbitrarily small, according to Equation (13), there exists $\tilde{\varepsilon }$ such that $\tilde{\varepsilon }/h_{N,T}<1$. In this case, $\hat{m}$ falls into $U\left(m_{0},\tilde{\varepsilon }/h_{N,T}\right)$ with an approximation to 1 in probability. According to the discreteness of $\hat{m}$, the probability of $\hat{m}$ is approximately equal to 1. □

## 4. Monte Carlo Simulations

For our simulations, we used the model

$$y_{it}=a_{i}+x_{i,t}^{\prime }\left(\beta +\Delta I\left\{t>k\right\}\right)+\varepsilon_{i,t},\;i=1,\cdots ,N,t=1,\cdots ,T,$$

with $x_{i,t}$ follow a uniform distribution U(5,8), and $a_{i}$ follow a uniform distribution U(1,3). The error term $\varepsilon_{it}$ is assumed to follow a standard normal distribution N(0,1).

We employ Monte Carlo simulations to assess the effectiveness of the JIC method. Specifically, we analyze the accuracy of the JIC test across different combinations of (N,T) and varying assumptions about $k^{*}$. Our simulation design explicitly includes scenarios with small *N* and *T* to investigate the small-sample properties of JIC.

Additionally, we compare the JIC method’s performance with the Wald-type approach proposed in [9], which utilizes a wild bootstrap procedure to derive critical values. All subsequent Monte Carlo experiments are repeated 1000 times. To compare the computational complexity of the methods, we also output the program runtime.

The choice of tuning parameters may affect the effectiveness of the method proposed in this paper. Therefore, Section 4.1 first discusses the selection of tuning parameters.

### 4.1. Choosing the Tuning Parameter γ

To ensure the effectiveness of the method, the selection of γ is data-driven. Based on simulation results, we provide a suitable range for γ in practical applications. First, for fixed sample sizes *N* and *T*, we evaluate the performance of change point estimation across different values of γ, identifying the range of γ that maximizes estimation accuracy. Next, this analysis is repeated under varying *N* and *T* to derive optimal γ intervals for each sample size configuration. Finally, the intersection of these intervals is taken to determine a recommended range for γ. To assess the proposed method’s robustness in tuning parameter selection, we conduct simulation experiments using the panel data model specified in (1) across diverse scenarios.

**Figure 1:**
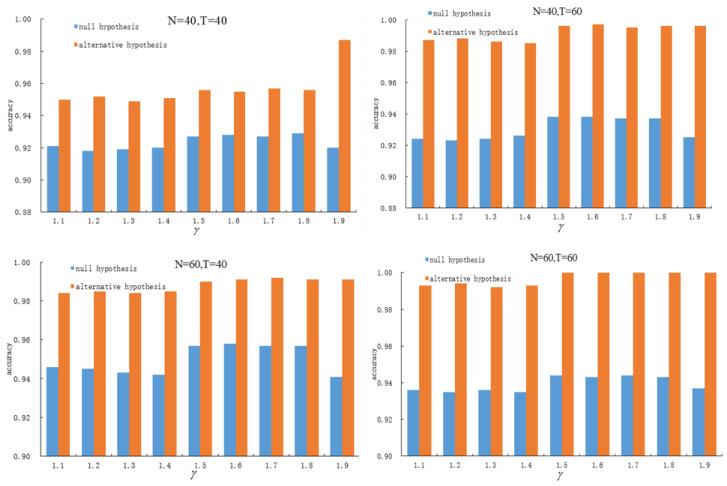
Accuracy of change point detection for different values of γ.

The magnitude of the structural break is set to Δ=0.2. We consider different combinations of cross-sectional units N=40,60 and time periods T=40,60 to compare the stability and estimation accuracy of the method in selecting γ, under both the null hypothesis and the alternative hypothesis. As shown in Figure 1, the accuracy of change point estimation becomes stable when γ falls within the interval 1.5≤γ≤1.8. Thus, when γ∈[1.5,1.8], the JIC estimation method achieves a balance between detecting zero and one change points.

### 4.2. Simulations

To evaluate the statistical efficiency of the jump information criterion under both the change-point and no-change-point scenarios, we set Δ=0.2,0.3, and 0.5, respectively. Let γ=1.6, and consider

$$P\left(N,T\right)=\frac{{\left({\hat{\beta }}_{1}-{\hat{\beta }}_{2}\right)}^{\prime }{\left[{\left({\hat{W}}_{xx}^{1}\right)}^{-1}+{\left({\hat{W}}_{xx}^{2}\right)}^{-1}\right]}^{-1}\left({\hat{\beta }}_{1}-{\hat{\beta }}_{2}\right)\cdot (N\cdot ∥\hat{\Delta }{∥)}^{\gamma }}{NT\left[N^{-\gamma }T^{\frac{\gamma }{2}}\cdot (N\cdot ∥\hat{\Delta }{∥)}^{\gamma }+1\right]}$$

Table 1 shows that under the null hypothesis (no change), the JIC method outperforms the Wald-type method in detection accuracy, even with small sample sizes. Table 2 presents results under the alternative hypothesis $H_{1}$, where the change point is set at k=T/2 with varying jump sizes. The JIC method achieves higher accuracy when the jump size is small. As the sample size and jump size increase, both methods reach 100% accuracy. In terms of computational efficiency, the JIC method is faster than the Wald-type method, especially for large samples. Furthermore, the experimental results confirm that the accuracy of estimating the true number of change points $m_{0}$ with the JIC method improves as *N* and *T* increase, supporting the conclusion of Theorem 1.

To examine the impact of the change point being located near the beginning or end of the sample on the accuracy of change point detection, we conducted additional simulation experiments under the alternative hypothesis $H_{1}$, setting the change point *k* at T/4 and 3T/4, respectively. The results are presented in Table 3. The findings indicate that when the change point is close to the beginning or end of the sample, the accuracy of change point detection is barely affected. Moreover, as the sample sizes *N* and *T* increase, the detection accuracy of the JIC method can also reach 100%.

To further examine the applicability and robustness of the proposed method under non-Gaussian settings, we supplement simulations in which the error term follows a heavy-tailed distribution, $\varepsilon_{it}$∼t(5). In this experiment, all other settings remain unchanged: $x_{i,t}$∼$U\left(5,8\right),a_{i}$∼U(1,3), and Δ=0.2,0.3,0.5.

The simulation results are in Table 4 and Table 5 and indicate that when the error term follows a t(5) distribution, the change-point detection accuracy slightly decreases compared with the Gaussian case N(0,1). For example, when N=50, T=50, and Δ=0.2, the detection accuracy is 0.978 under normal errors, whereas it is 0.926 under the t(5) distribution.

Although the Wald-type method exhibits slightly higher detection accuracy than the JIC method when Δ=0.2, the accuracy of the JIC method also reaches 100% as the sample sizes *N* and *T* and the change magnitude Δ increase. In terms of computational efficiency, the JIC method is generally faster than the Wald-type method, especially for large samples, because it avoids constructing test statistics and choosing significance levels, thereby reducing computation time.

This phenomenon is due to the heavier tails of the t(5) distribution, which generate more extreme values, increasing random fluctuations and making it more difficult to distinguish structural changes from random noise. Nevertheless, as the sample sizes *N* and *T* and the change magnitude Δ increase, the JIC method improves in estimating the true number of change points $m_{0}$ and in achieving higher overall detection accuracy. The trend is consistent with that under Gaussian errors, indicating that the JIC method maintains good applicability and robustness under non-Gaussian errors.

### 4.3. An Empirical Application

**Figure 2:**
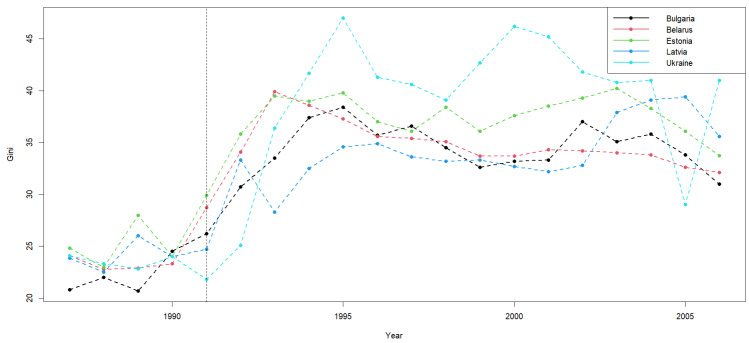
Time series of Gini coefficients (percentage) in five former socialist countries.

To demonstrate the relevance of our findings, we drew on data from the World Income Inequality Database, accessible at https://www.wider.unu.edu/project/world-income-inequality-database, (accessed on 21 March 2026). The primary dataset comprises Gini coefficients in percentage points, along with source details, encompassing 159 countries (the Gini index is used to measure the inequality of wealth). The original dataset comprises Gini coefficients in percentage points and source details for 159 countries. Unfortunately, a significant amount of data is missing, especially predating the 1980s. We therefore selected five countries from 1987 to 2006, including Belarus, Bulgaria, Estonia, Latvia, and Ukraine. Each country has a minimum of 16 recorded observations (Figure 2). Missing observations were replaced using linear interpolation. If more than one point was given for one year, they were replaced by the average. Therefore, our analysis is based on N=5 and T=20.

We applied the method proposed in this paper and the methods in the literature to conduct change point analysis on this dataset.

First, using our method, the calculated JIC values are JIC(1)=37.6368, JIC=8.8626. Since JIC(1)<JIC(0), the estimated number of change points is $\hat{m}=1$. The estimated change point location is ${\hat{k}}^{*}=5$. The total computation time for change point analysis is 0.014 s.

Second, using the methods in the literature, a Wald-type test was first performed, which indicated the presence of a change point. The CUSUM method was then used to estimate the change point location, yielding ${\hat{k}}^{*}=6$. The total computation time for this approach was 0.021 s.

These results clearly demonstrate the advantages of our method, particularly in change point analysis for massive datasets, where its efficiency gains will be even more pronounced.

## 5. Conclusions

For the problem of detecting a single change point in linear panel data models, this paper proposes an estimation method based on the jump information criterion (JIC). By framing the hypothesis testing problem as an estimation problem for the number of change points, the method obviates the need for restrictive assumptions about nuisance parameters or data distributional forms. By minimizing the JIC, an estimate of the change point count is derived. The paper establishes the consistency of this estimate and characterizes its optimal convergence rate. Monte Carlo simulations confirm the method’s effectiveness in correctly maintaining the null hypothesis (no change point) and achieving high estimation accuracy under the alternative hypothesis (one change point). This transformation brings several advantages, which we elaborate as follows:

(1) Simplified Procedure: The JIC method estimates the number of change points by minimizing an information criterion, without requiring the construction of test statistics or the selection of significance levels. This makes the procedure more intuitive, practical, and user-friendly.
(2) Reduced Computational Burden: Compared to hypothesis testing approaches, JIC eliminates the need for additional steps such as computing critical values or bootstrap distributions. As a result, the method significantly reduces computation time and operational complexity, especially for large datasets.
(3) High Detection Accuracy with Small Changes: Simulation results show that even when the magnitude of structural changes is small, the JIC method maintains a high level of detection accuracy, which demonstrates its robustness in practical applications.

Notably, while the proposed JIC framework is scalable to scenarios involving multiple change points, high-dimensional settings, nonstationary series, integrated (cointegrated) data, and panel data models with latent group structures, inherent challenges arise in such generalizations. Ongoing research is dedicated to addressing these multifaceted complexities, including refining penalty terms to accommodate group-specific dynamics, enhancing computational efficiency, and refining the methodology to achieve broader applicability across these diverse empirical contexts. For the high-dimensional change point problem, Wang and Samworth (2018) [16] proposed a two-stage procedure called INSPECT for estimating change points. In future research, we will focus on applying the JIC method to change point analysis in high-dimensional data.

## Figures and Tables

**Table 1:** Under the null hypothesis, there is no change in the coefficients; the accuracy of ${\hat{m}}_{0}=m_{0}$ is obtained by two competing methods under a Gaussian distribution.

N/T		JIC	Wald-Type
50/50	accuracy	0.964	0.942
	time/s	8.43	8.76
100/50	accuracy	0.975	0.963
	time/s	9.56	9.87
100/100	accuracy	0.966	0.957
	time/s	11.62	12.56
200/100	accuracy	0.985	0.973
	time/s	14.31	15.89

**Table 2:** When the change is located at k=T/2 with coefficient shifts (Δ=0.2,0.3 and 0.5); we compare the accuracy of ${\hat{m}}_{0}=m_{0}$ for the two competing methods under a Gaussian distribution.

N/T		Δ=0.2		Δ=0.3		Δ=0.5	
		**JIC**	**Wald-Type**	**JIC**	**Wald-Type**	**JIC**	**Wald-Type**
50/50	accuracy	0.978	0.969	1	1	1	1
	time/s	8.56	8.87	8.55	8.86	8.55	8.85
100/50	accuracy	0.993	0.988	1	1	1	1
	time/s	9.67	9.95	9.65	9.97	9.66	9.96
100/100	accuracy	1	1	1	1	1	1
	time/s	11.87	12.75	11.86	12.74	11.85	12.73
200/100	accuracy	1	1	1	1	1	1
	time/s	14.52	15.92	14.51	15.93	14.53	15.93

**Table 3:** When the change is located at k=T/4 or k=3T/4 with coefficient shifts (Δ=0.2,0.3 and 0.5); we compare the accuracy of ${\hat{m}}_{0}=m_{0}$ for the two competing methods under a Gaussian distribution.

N/T		Δ=0.2		Δ=0.3		Δ=0.5	
		**JIC**	**Wald-Type**	**JIC**	**Wald-Type**	**JIC**	**Wald-Type**
$k^{*}=T/4$							
50/50	accuracy	0.967	0.963	1	1	1	1
	time/s	8.58	8.88	8.57	8.86	8.56	8.86
100/50	accuracy	0.988	0.982	1	1	1	1
	time/s	9.68	9.96	9.66	9.97	9.66	9.98
100/100	accuracy	1	1	1	1	1	1
	time/s	11.88	12.74	11.87	12.73	11.86	12.73
200/100	accuracy	1	1	1	1	1	1
	time/s	14.53	15.94	14.52	15.95	14.54	15.96
$k^{*}=3T/4$							
50/50	accuracy	0.958	1	1	1	1	1
	time/s	8.59	8.89	8.57	8.88	8.56	8.87
100/50	accuracy	0.991	1	1	1	1	1
	time/s	9.69	9.97	9.67	9.98	9.66	9.97
100/100	accuracy	1	1	1	1	1	1
	time/s	11.88	12.75	11.87	12.74	11.86	12.73
200/100	accuracy	1	1	1	1	1	1
	time/s	14.52	15.93	14.53	15.94	14.54	15.94

**Table 4:** When the change is located at $k^{*}=T/2$ with coefficient shifts (Δ=0.2,0.3 and 0.5); we compare the accuracy of ${\hat{m}}_{0}=m_{0}$ for the two competing methods under a t(5) distribution.

N/T		Δ=0.2		Δ=0.3		Δ=0.5	
		**JIC**	**Wald**	**JIC**	**Wald**	**JIC**	**Wald**
50 / 50	accuracy	0.926	0.953	1	1	1	1
	time/s	8.63	8.92	8.64	8.9	8.61	8.89
100 / 50	accuracy	0.973	0.979	1	1	1	1
	time/s	9.71	10.11	9.69	10.08	9.62	10.07
100 / 100	accuracy	1	1	1	1	1	1
	time/s	11.98	12.79	11.97	12.76	11.89	12.74
200 / 100	accuracy	1	1	1	1	1	1
	time/s	14.66	15.98	14.63	15.83	14.51	15.82

**Table 5:** When the change is located at $k^{*}=T/4$ or $k^{*}=3T/4$ with coefficient shifts (Δ=0.2,0.3 and 0.5), we compare the accuracy of ${\hat{m}}_{0}=m_{0}$ for the two competing methods under a t(5) distribution.

N/T		Δ=0.2		Δ=0.3		Δ=0.5	
		**JIC**	**Wald**	**JIC**	**Wald**	**JIC**	**Wald**
$k^{*}=T/4$							
50/50	accuracy	0.889	0.941	1	1	1	1
	time/s	8.68	8.99	8.65	8.97	8.65	8.96
100/50	accuracy	0.943	0.988	1	1	1	1
	time/s	9.77	9.82	9.77	9.81	9.76	9.81
100/100	accuracy	1	1	1	1	1	1
	time/s	11.99	12.78	11.96	12.78	11.96	12.77
200/100	accuracy	1	1	1	1	1	1
	time/s	14.68	16.11	14.66	16.12	14.66	16.11
$k^{*}=3T/4$							
50/50	accuracy	0.865	0.997	0.998	1	1	1
	time/s	8.69	8.99	8.64	8.97	8.63	8.92
100/50	accuracy	0.931	1	1	1	1	1
	time/s	9.77	9.83	9.77	9.81	9.76	9.81
100/100	accuracy	1	1	1	1	1	1
	time/s	11.96	12.79	11.96	12.77	11.95	12.76
200/100	accuracy	1	1	1	1	1	1
	time/s	14.69	16.09	14.66	16.11	14.66	16.09

## Data Availability

The original contributions presented in this study are included in the article. Further inquiries can be directed to the corresponding author.
